# Is public transport a promising strategy for increasing physical activity? Evidence from a study of objectively measured public transport use and physical activity

**DOI:** 10.1186/s12966-024-01633-3

**Published:** 2024-08-19

**Authors:** Jack. T. Evans, Oliver Stanesby, Leigh Blizzard, Stephen Greaves, Anna Timperio, Kim Jose, Melanie J. Sharman, Andrew J. Palmer, Verity J. Cleland

**Affiliations:** 1 Menzies Institute for Medical Research, University of Tasmania, 17 Liverpool St, Hobart, Tasmania 7000 Australia; 2 Baker Heart and Diabetes Institute, Melbourne, Australia; 3 Institute of Transport and Logistics Studies, University of Sydney, Sydney, Australia; 4 Institute for Physical Activity and Nutrition, School of Exercise and Nutrition Sciences, Deakin University, Geelong, Australia

**Keywords:** Commuting, Exercise, Public health, Transportation, Translational medical research, Behaviour

## Abstract

**Background:**

Greater public transport use has been linked to higher physical activity levels. However, neither the amount of physical activity associated with each daily public transport trip performed, nor the potential total physical activity gain associated with an increase in trips/day, has been determined. Using objective measures, we aimed to quantify the association between public transport use, physical activity and sedentary time.

**Methods:**

A longitudinal study of Australian adults living in Hobart, Tasmania, who were infrequent bus users (≥ 18 years; used bus ≤ 2 times/week). The number of bus trips performed each day was determined from objective smartcard data provided by the public transportation (bus) provider across a 36-week study timeframe. Accelerometer measured steps/day (primary outcome), moderate-to-vigorous physical activity (min/day), and sedentary time (min/day) were assessed across four separate one-week periods.

**Results:**

Among 73 participants across 1483 day-level observations, on days that public transport was used, participants achieved significantly more steps (β = 2147.48; 95%CI = 1465.94, 2829.03), moderate to vigorous physical activity (β = 22.79; 95% CI = 14.33, 31.26), and sedentary time (β = 37.00; 95% CI = 19.80, 54.21) compared to days where no public transport trips were made. The largest increase in steps per day associated with a one-trip increase was observed when the number of trips performed each day increased from zero to one (β = 1761.63; 95%CI = 821.38, 2701.87). The increase in the number of steps per day was smaller and non-significant when the number of trips performed increased from one to two (β = 596.93; 95%CI=-585.16, 1779.01), and two to three or more (β = 632.39; 95%CI=-1331.45, 2596.24) trips per day. Significant increases in sedentary time were observed when the number of trips performed increased from zero to one (β = 39.38; 95%CI = 14.38, 64.39) and one to two (β = 48.76; 95%CI = 25.39, 72.12); but not when bus trips increased from two to three or more (β=-27.81; 95%CI=-76.00, 20.37).

**Conclusions:**

Greater public transport use was associated with higher physical activity and sedentary behaviour. Bus use may yield cumulative increases in steps that amount to 15–30% of the daily recommended physical activity target. A policy and public health focus on intersectoral action to promote public transport may yield meaningful increases in physical activity and subsequent health benefits.

**Supplementary Information:**

The online version contains supplementary material available at 10.1186/s12966-024-01633-3.

## Background

One in four adults globally do not meet the World Health Organization minimum recommendation of 150 min of moderate physical activity each week [1]. Given physical inactivity’s association with increased risk of chronic diseases and early mortality [2], the promotion of physical activity and achievement of guidelines is crucial for public health. Due to the need for transit from one place to another, transport-related physical activity has been identified as a behaviour in which overall physical activity levels may be increased [3, 4]. The contribution of public transport use towards transport-related physical activity, and subsequently total physical activity levels is primarily observed via incidental walking and cycling to and from public transport access terminals. A systematic review conducted in 2012 of 27 studies drawn largely from the United States and United Kingdom reported the additional physical activity (walking and cycling) associated with using public transportation ranged from 8-33 min/day, with most studies reporting 12-15 min/day [5]. Higher physical activity levels have been reported among public transport users in Victoria, Australia (additional 33 min/day) and the United States (additional 30 min/day) [6, 7]. Similarly, undertaking trips via public transport may yield greater walking and shorter sitting durations than trips undertaken via private motor vehicle [8].

Most studies examining the relationship between public transport use and physical activity have relied on self-report diaries and retrospective surveys to assess one or both behaviours [5, 9–12]. The use of subjective measurement tools (e.g., self-report) for the assessment of exposures and/or outcomes has the potential to reduce internal validity through recall bias, social desirability bias, and differential reporting of physical activity behaviours and public transportation use. Conversely, objectively assessed physical activity (via accelerometer and other devices) and public transport use (via smartcard trip logs) can provide more accurate information and insights into both behaviours and can subsequently facilitate a more accurate and reliable examination of the relationship between public transport use and physical activity. Despite the advantages, there is a scarcity of studies examining the relationship between public transport use and physical activity using objective measures. Furthermore, prior analyses of public transportation use and physical activity that used objective measures (physical activity or public transport or both) have primarily been performed at the week-level (or greater), reporting increased activity among public transport users verses private transport users [5]. However, week-level analysis, rather than examination at the day-level, overlooks the variability of daily bus use and physical activity, thus, providing less detailed and less precise findings. To our knowledge, no examination of the relationship between objective measures of physical activity, sedentary behaviour, and public transport at the day-level have been published.

This study aimed to address these knowledge gaps by quantifying the association between objectively measured public transport trips and total daily ambulatory physical activity (steps), moderate-to-vigorous physical activity (MVPA) and sedentary time. It was hypothesised that (a) greater physical activity and lower sedentary time would be observed on days public transport was used, compared to those on which it was not, and (b) physical activity would continue to increase and sedentary time decrease as more public transport trips were performed each day (i.e., a dose-response association).

## Methods

### Study design and participants

This longitudinal study comprised adults (≥ 18 years) residing in the Tasmanian state capital, the Greater Hobart region, Australia [13], who infrequently used public transport (≤ 2 trips per week) and were recruited as part of the COVID-19-abandoned *trips4health* randomised control trial [14–17]. Participants were recruited using bus advertising, word-of-mouth, social and traditional media promotion, and workplace and professional networks.

Tasmania (population 550,000) is a mostly regional state with four main cities, the largest being the capital Hobart (population 253,000) [18]. In Hobart in 2019-20, bus was the only public transport service available, provided primarily in urban areas by a single provider. The *trips4health* study originally planned to assess the impact of a four-month financial incentive-based program to increase public transport use, with public transport use and physical activity measurement at three timepoints (T1: 0 months, T2: 4 months, T3: 10 months) among a control group and an intervention group [14].

The *trips4health* randomised control trial was abandoned after approximately one third of the target sample had been recruited due to the COVID-19 pandemic; a state of emergency was declared by the Tasmanian government and subsequent social restrictions were enacted (detailed elsewhere [14, 16, 17]), limiting study participation. When the *trips4health* study was abandoned, 110 participants had completed the T1 assessment, 64 of whom had completed the T2 assessment, and none had completed the T3 assessment. All participants that completed the T1 assessment of the *trips4health* study, irrespective of T2 completion, were invited to complete two additional assessments following randomised control trial abandonment (T3: from July 3rd, 2020; then T4: three months later). The control group and intervention group were combined for this analysis.

### Ethics, consent, and permissions

**Fig. 1:**
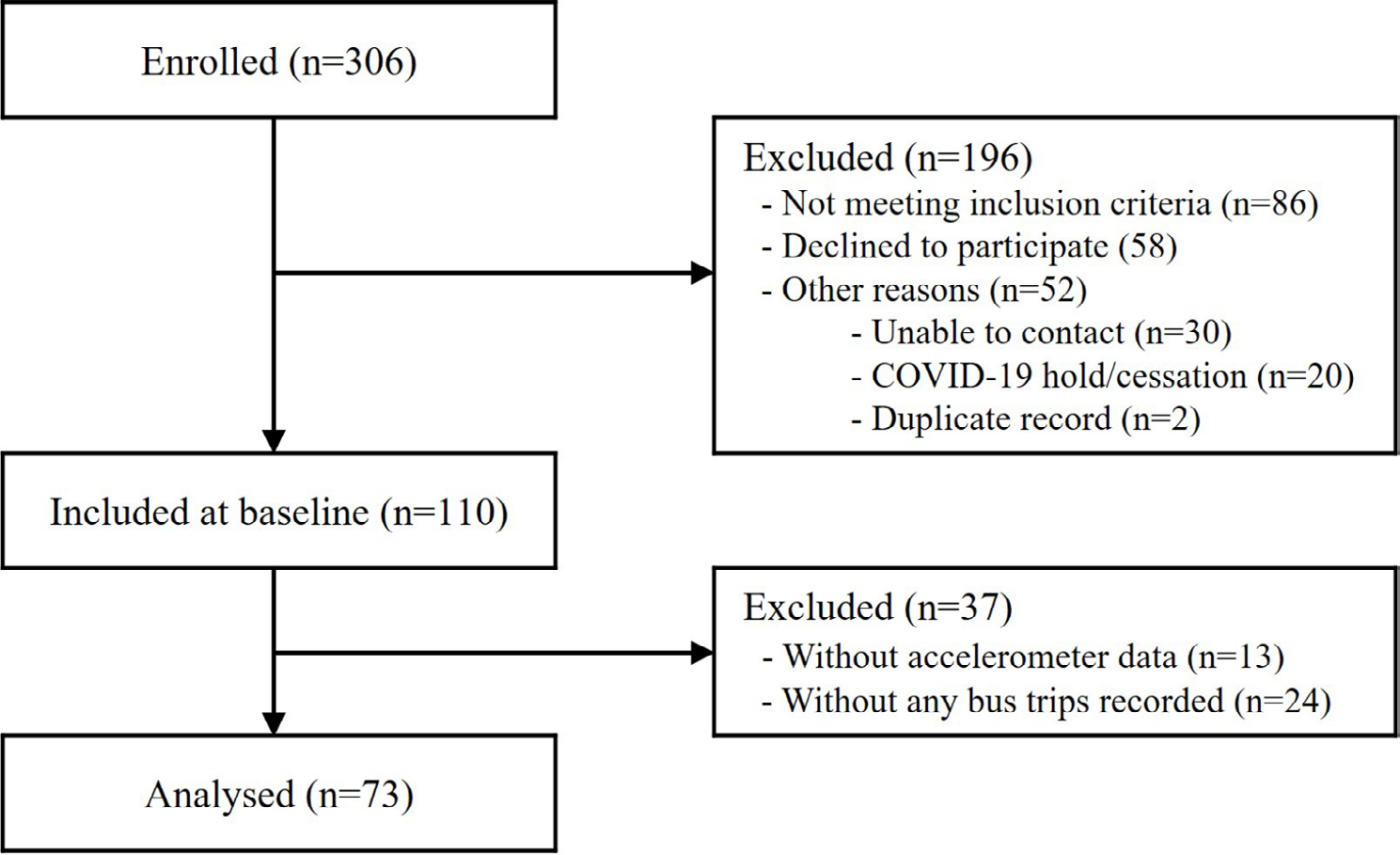
Flowchart of participant inclusion

Ethics approval was granted by the Tasmanian Health and Medical Human Research Ethics Committee (approval number H0017820, 27 March 2019). The *trips4health* study was registered with the Australian and New Zealand Clinical Trials Registry August 14th, 2019: ACTRN12619001136190; *Universal Trial Number*: U1111-1233-8050. Participant consent was provided electronically through REDCap (Research Electronic Data Capture, Version 8.5.19, Nashville, Tennessee, USA) or in person with research staff when attending the first clinic (T1). A STROBE (strengthening the reporting of observational studies in epidemiology) checklist is presented in the supplementary material (Additional file 1, Table S1) and flowchart of participant inclusion is presented in Fig. 1.

### Outcome

The primary outcome was daily step count (total ambulatory activity), secondary outcomes were total daily MVPA and total daily sedentary time. Outcomes were assessed via ActiGraph GT3X accelerometers worn on the right hip, during waking hours, for seven days at T1, T2, T3, and T4. Daily step count (steps/day), MVPA (minutes/day), and sedentary time (minutes/day) were then derived using ActiLife v6.13.3 analysis software (ActiGraph, Pensacola, FL, USA). Monitor wear time was determined by manual examination of wear log information and the 2011 Choi et al. [19] automated wear time estimate. A default 60-second epoch was observed. Accelerometer data was included if the participant had at least eight hours of valid wear time for a minimum of three days, consistent with prior research evaluating accelerometer reliability [20] and suggestions of Trost et al. [21].

Total MVPA and sedentary time were determined using the 1998 Freedson metabolic cost regression equation, giving the following cut points: sedentary time (0–99 counts per minute); MVPA (≥ 3 METs / ≥1952 counts per minute) [22].

### Exposure

The number of bus trips made by each participant on each day they wore an accelerometer was derived from Metro Tasmania smartcard travel data. Bus trips were recorded at boarding, when participants recorded a fare using their smartcard on the bus’ terminal. Each ‘bus trip’ was comprised of an independent single, or sequential series of trips that were performed within a 90-minute connecting period.

### Participant characteristics

At T1, participants completed an online questionnaire: *Age* (years) was derived from date of birth. *Gender* was reported as man, woman, trans, or other. *Highest level of education* was categorised as: low (year 12 or less); medium (trade/apprenticeship, certificate/diploma); and high (university degree, higher degree). *Employment* status was categorised as: full time; part time; not employed/working. The *number of dependents* (children under 15 years old) living in the household was reported. *Marital status* was categorised as: married (living in a registered marriage/a de facto relationship); and not married (separated, divorced, widowed; never married). *Self-rated health* was categorised into three levels: excellent/very good, good, and fair/poor. *Smoking status* was reported as: current smoking status (yes or no).

At T1, the following measures were assessed in clinic: Participants’ *height* was measured to the nearest 0.1 cm via fixed stadiometer (93% MedTec, 7% Charder). *Weight* was assessed to the nearest 0.1 kg using electronic UC-321PL Precision Scales (94% A & D Medical, 6% Heine). *Body mass index* was calculated using weight (kg)/height (m)^2^.

### Statistical analysis

Analysis was performed using STATA version 17.0 (StataCorp LP, College Station, Texas, USA). Participant characteristics were presented as means and standard deviations (SD) for continuous variables and as percentages and frequencies for categorical variables. Steps per day and MVPA were presented as median and interquartile range (IQR); sedentary time was presented as mean (SD). Complete-case analysis was performed using observations at any of the four timepoints with both public transport trip and accelerometer data. Multi-level mixed effects modelling was performed to assess the relationship between number of bus trips and the primary outcome of total steps and secondary outcomes of MVPA and sedentary time, while accounting for within-individual correlation of the 1483 day-level observations sourced from 73 participants. The outcome of steps/day was square-root transformed and modelling completed; coefficients were then back-transformed. Model 1 was performed unadjusted for covariates, model 2 was performed adjusting for baseline covariates. Covariates (confounders) included in model 2 were informed via a review of the existing literature and retained in final models based on the presence of statistical significance of their association with both the exposure of interest and the outcome, and whether the estimated coefficient of the relationship between exposure and outcomes changed by more than 10% when potential covariates were included/excluded. Two additional sensitivity analyses were performed, testing for interactions by gender and by randomised control trial treatment group.

## Results

### Participant characteristics

Seventy-three participants, representing 1483 observations (participant-days), were included in this analysis. A STROBE flowchart of participant inclusion is presented in Fig. 1. The mean baseline (T1) age of participants was 45.2 years. Mean body mass index was 27.8 kg/m^2^ and 27.4% of participants were employed full-time. Approximately 50% of *Trips4health* participants and Greater Hobart Bus users were classified as having high-levels of education; and 61% were women [23]. Participant age, employment, and marital status more closely resembled the characteristics of the Greater Hobart general populace. Further participant characteristics are presented in Table 1. The distribution of daily trips recorded at each timepoint are shown at the person and day level (days observed) in Table S2 and shown further stratified by treatment group in Table S3.

**Table 1:** Baseline characteristics of study and Greater Hobart populations

	trips4health	Greater Hobart bus users*	Greater Hobart total*
N	73	6679	197,357
Age (years), mean (SD)	45.2 (16.9)	37.0 (13.1)	48.4 (19.0)
Gender (female), % (n)	61.6 (45)	60.8 (4060)	51.9 (102,393)
Body mass index (kg/m^2^), mean (SD)	27.8 (6.3)		
**Current smoker, % (n)**			
Yes	6.8 (5)		
No	93.2 (68)		
**Self-rated health, % (n)**			
Excellent/very good	42.5 (31)		
Good	43.8 (32)		
Fair/poor	13.7 (10)		
**Education level, % (n)**			
Low	20.6 (15)	28.6 (1833)	38.3 (68,364)
Medium	30.1 (22)	21.6 (1380)	28.1 (50,224)
High	49.3 (36)	49.8 (3187)	33.6 (59,913)
**Employment status, % (n)**			
Full-time (> 35 h/week)	27.4 (20)	53.3 (3461)	35.7 (64,974)
Part-time (1–34 h/week)	41.1 (30)	46.7 (3037)	24.4 (44,345)
Unemployed (0 h/week)	31.5 (23)	0.0 (0)	39.9 (72,541)
**Marital status, % (n)**			
Married/de facto	59.1 (39)	44.7 (2938)	53.1 (104,739)
Not married/de facto	40.9 (27)	55.3 (3689)	46.9 (92,620)
Number of dependents, mean (SD)	2.0 (0.67)		
Bus trips/day, mean (SD)	0.31 (0.74)		
Steps/day, median (IQR)	7010 (4675, 9943)		
MVPA (min/day), median (IQR)	37 (15, 62)		
Sedentary time (min/day), mean (SD)	529.1 (155.1)		

IQR = Inter quartile range, MVPA = moderate-vigorous physical activity, SD = Standard deviation. N of Greater Hobart region varies as shown

Greater Hobart bus users depicts individuals that used public transport (buses) to commute to work on census day

*Greater Hobart data source: Census of Population and Housing, 2021, TableBuilder: https://www.abs.gov.au/statistics/microdata-tablebuilder/tablebuilder Copyright Commonwealth of Australia, 2021, see abs.gov.au/copyright. ABS data licensed under Creative Commons, see abs.gov.au/ccby

### Bus trips and physical activity

Compared to days with fewer bus trips, participants had a greater number of steps on days with a greater number of bus trips in both unadjusted and adjusted models that account for intra-individual covariance (Table 2). Compared to days where no bus trips were made, on days with one or more bus trips participants accumulated on average 2147.5 more steps (β = 2147.48; 95% CI = 1465.94, 2829.03), 22.8 more minutes of MVPA (β = 22.79; 95% CI = 14.33, 31.26), and 37.0 min more sedentary time (β = 37.00; 95% CI = 19.80, 54.21).

**Table 2:** Change in steps/day, moderate to vigorous physical activity, and sedentary time for each additional bus trip taken

	Model 1 β (95% CI)	Model 2 β (95% CI)
**Total ambulatory physical activity (steps/day)**		
0 to ≥ 1 bus/trips/day	**1661.08 (1184.00**,** 2138.17)**	**2147.48 (1465.94**,** 2829.03)**
0 to 1 bus trip/day	**1342.34 (656.29**,** 2028.40)**	**1761.63 (821.38**,** 2701.87)**
1 to 2 bus trips/day	466.69 (-441.23, 1374.62)	596.93 (-585.16, 1779.01)
2 to ≥3 bus trips/day	468.08 (-1052.85, 1989.01)	632.39 (-1331.45, 2596.24)
**Moderate to vigorous physical activity (mins/day)**		
0 to ≥ 1 bus/trips/day	**14.38 (9.96**,** 18.80)**	**22.79 (14.33**,** 31.26)**
0 to 1 bus trip/day	**10.23 (4.04**,** 16.41)**	**16.34 (5.91**,** 26.77)**
1 to 2 bus trips/day	7.29 (-1.09, 15.69)	11.28 (-2.05, 24.60)
2 to ≥ 3 bus trips/day	-0.55 (-14.57, 13.47)	-0.62 (-22.29, 21.04)
**Total sedentary time (mins/day)**		
0 to ≥ 1 bus/trips/day	**36.53 (19.33**,** 53.73)**	**37.00 (19.80**,** 54.21)**
0 to 1 bus trip/day	**38.43 (13.44**,** 63.43)**	**39.38 (14.38**,** 64.39)**
1 to 2 bus trips/day	**48.55 (25.18**,** 71.92)**	**48.76 (25.39**,** 72.12)**
2 to ≥ 3 bus trips/day	-27.57 (-75.74, 20.61)	-27.81 (-76.00, 20.37)

β = coefficient representing the increase in each outcome variable for each additional bus trip undertaken. 95% CI = 95% confidence interval

Bold text indicates statistical significance

Model 1 = unadjusted

Model 2 = adjusted for age, gender, body mass index, education, and employment

**Fig. 2:**
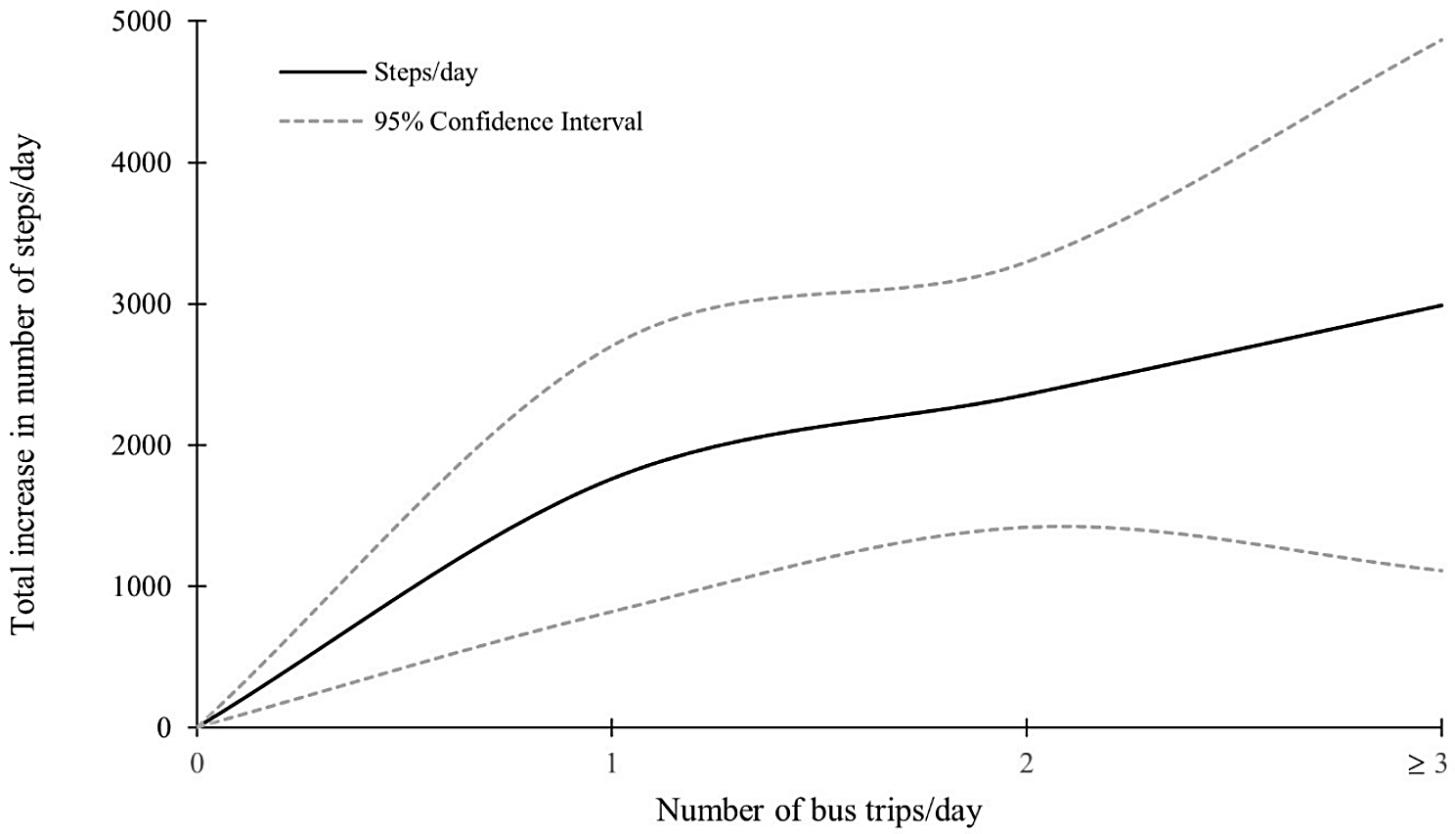
Summative increase in number of steps with each additional bus trip undertaken. Total increase in number of steps taken with each additional bus trips performed per day, adjusted for covariates, with 95% confidence intervals

For each single trip increase in bus trips (i.e., 0 to 1, 1 to 2, 2 to ≥ 3), steps/day, MVPA, and sedentary time increased, with associations strengthening with adjustment for covariates of age, gender, body mass index, education, and employment (Table 2).

Compared to days when no bus trips were taken, on days when they made one bus trip participants recorded 1761.6 more steps (β = 1761.63; 95% CI = 821.38, 2701.87) and 16.3 more minutes of MVPA (β = 16.34; 95% CI = 5.91, 26.77) (Table 2). Sedentary time was also greater on days when the number of daily bus trips increased from zero to one (β = 39.38; 95% CI = 14.38, 64.39).

On days when they made two bus trips, participants recorded 596.9 (β = 596.93; 95% CI= -585.16, 1779.01) more steps (non-significant) and 11.3 more minutes of MVPA (β = 11.28; 95% CI= -2.05, 24.60) (non-significant) than on days they took one bus trip (Table 2). Sedentary time was higher by 48.8 min (β = 48.76; 95% CI = 25.39, 72.12) on days when the number of daily bus trips increased from one to two. Taking the bus twice in a day was associated with 2358.5 more steps than on days with no bus travel (β = 2358.55; 95% CI = 1420.74, 3296.37) (Fig. 2).

On days when they made three or more bus trips, participants completed 632.4 more steps (β = 632.39; 95% CI= -1331.45, 2596.24) (non-significant) and performed 0.6 min less MVPA (β= -0.62; 95% CI= -22.29, 21.04) (non-significant) than on days they took two bus trips (Table 2). However, no significant difference in sedentary time was observed when undertaking ≥ 3 bus trips compared to two (β= -27.8; 95%CI = -76.00, 20.37) (Table 2).

2990.9 more steps were recorded on days which three or more bus trips were taken, compared to days participants did not catch the bus (β = 2990.95; 95% CI = 1112.87, 4869.02) (Fig. 2); however, these observations must be interpreted with caution due to limited observations with more than three bus trips per day (*n* = 12 (0.67%) of total observations).

### Sensitivity analyses

There were no significant interactions by gender (*p* = 0.29) or treatment group (control/intervention) (*p* = 0.09) when assessed via Wald test. Stratified analysis by treatment group was performed, however statistically significant differences were not observed - confidence intervals were extremely wide, overlapped considerably between treatment and control groups, and included the null.

## Discussion

This study aimed to quantify the association between objectively measured daily bus use, physical activity, and sedentary time. On days where participants made more bus trips, they took more steps and accumulated more MVPA, but were more sedentary. Cumulative increases in steps/day between 1500 and 3000 steps/day provide important contributions (15–30%) towards often-promoted physical activity goals of 10,000 steps/day. While other studies have observed positive relationships between public transport and physical activity [24], to our knowledge, this is the first to examine the relationship between device-assessed physical activity and smartcard-recorded public transport use [9–12]. Further, this is also the first to assess these associations at the day, rather than per week (or greater) level, providing more detailed insights into the relationship between public transport use and physical activity.

On average, a single bus trip was associated with 1761.6 more steps for that day compared to a day in which the bus was not used at all. With each additional bus trip performed, increases in steps were observed but were not as large (e.g., 1761 to 597 step increase). The differing magnitudes of effect for each additional bus trip suggest a non-linear relationship between the number of bus trips and the number of additional steps taken each day. This may potentially be explained by differing walking routes and distances between points of embarkation and disembarkation for trips to and from destinations in the Greater Hobart region. For example, a participant may undertake a 1000 m walk from the home to a bus stop, a 500 m walk to work or place of study from the bus trip’s ending in the bus depot of Hobart’s central business district, then at the end of the day, repeat the same 500 m walk returning to the central bus depot, coupled with a bus trip that allows for disembarkation closer to the home, and a shorter 500 m walk. It is also plausible that participants use a different method of transport on one journey, for example, a bus trip to work and then a passenger in a vehicle on the way home.

Days involving a greater number of bus trips were associated with a higher number of total steps. While prior research has shown that the accumulation of 10,000 daily steps may yield health benefits [25], lower thresholds of activity (i.e., 1000–2000 steps) also result in reduction of all-cause mortality [26] as well as cardiovascular disease and cancer risk [27]. The findings of our study suggest that the promotion of public transport (bus) usage has the potential to provide a substantial contribution to the achievement of these daily physical activity goals. The increase in steps/day associated with the change from zero to one bus-trip/day (1761.6 steps) and from zero to any bus trips/day (2147 steps) is greater than the minimum threshold for clinically significant improvements in chronic pulmonary disease (350–1100 steps) [28, 29] and multiple sclerosis (1455 steps) [30].

Similarly, taking the bus was associated with more MVPA compared to days on which no public transport was used. Our finding of a 16-min/day difference in MVPA between days with no bus trips and days with one bus trip, and a 23-min/day difference between days with no bus trips and days with any number of bus trips, suggests that public transport use may contributed 10–15% towards the achievement of weekly physical activity guidelines. Increases in MVPA of this magnitude have previously been associated with decreased cardiovascular [31, 32] and all-cause mortality risk [33]. Moreover, a meta-analysis has shown similar curvilinear relationships to exist between daily steps and MVPA with outcomes of all-cause mortality [26]. As such, the steep early slope of these dose–response curves and low clinical thresholds for reduced disease risk [26], coupled with our findings, suggests that individuals with low physical activity volumes and no engagement with public transportation may experience the greatest health benefits from promotion of public transportation use.

Contrary to our hypothesis and prior studies [8], time in total sedentary behaviour increased with the number of bus trips performed. This relationship may potentially be explained by the greater time spent sitting for a trip undertaken via bus (with a specified route and required stops to pick up other passengers), compared to the time taken for the same trip, if performed using private motorised transport, on days of no or lower public transport use [34]. Further, it may be that public transport was primarily used on weekdays for work or study commutes [35, 36], on which days greater sedentary time may be accumulated at work among those with desk-based occupations, compared to non-work or work-from-home days on which no bus travel is performed. Theories of physical activity displacement [37], suggest increased sedentary time yields a net loss of time available for physical activity. Despite the reduction in potential time in which activity may take place (30 to 50 min reduction/day), a greater quantity of physical activity (increased number of steps/day) was performed in this study. This net increase in physical activity may act to alleviate clinical concerns of bus-related sedentary time.

### Strengths and limitations

This study had some limitations. Importantly, rising concerns regarding COVID-19 during the study period could have impacted both transport and physical activity behaviours, although it is likely participants were impacted non-differentially with regard to study-relevant variables. Prior analysis of this cohort found that during the period of peak COVID-19 restrictions in Tasmania, both public transport use and physical activity decreased but post-restrictions, physical activity returned to prior levels while public transport use remained lower [16]. Participants in this study were recruited as part of a randomised control trial to increase public transport use and allocated to a control and intervention group (receiving financial incentivisation to use public transport), however, treatment group did not meet criteria for confounding and both testing and stratified analyses observed no significant interaction to be present. A key strength of this study was its novel use of objective measures of physical activity and sedentary time and public transport usage. Accelerometers are an accurate and reliable objective measure of both step count and activity frequency, duration, and intensity [38, 39], thus allowing assessment of total ambulatory physical activity, sedentary time, and MVPA that is relatively free from information bias. The objective assessment of bus use via smartcard data provides detailed day-level information that allows the unbiased determination of number of trips, multi-leg journeys, and days and times of public transport use [40]. Furthermore, the use of smartcard data reliably captures day-to-day variability in public transport use that may be less reliable or overlooked when surveys are used. The external validity of this study to the broader population may be limited due to the over-representation of women and university educated participants in this sample, but is likely applicable to Hobart public transport users, as demonstrated through similarities of characteristics shown in Table 1. Further, as participants in this study volunteered to take part in a public transport intervention, it is possible they have differing motivations towards bus use than the general population. While this study was comprised of infrequent public transport users, the study’s low baseline rates of public transport-use are generally reflective of the greater Tasmanian population. As such, this study’s findings and recommendations of public transport promotion for physical activity gain may well be applicable to the greater population in which they were situated.

## Conclusions

This study found that daily steps were 29.2% and MVPA was 54.8% higher on days where public transport was used, amounts that could contribute significantly to reductions in cardiovascular disease, cancer, and multiple sclerosis risk. Greater daily public transport use were associated with further cumulative gains in physical activity (15–30% of the commonly promoted 10,000 step/day goals). Unexpectedly, significantly higher sedentary time were also observed with greater public transport use, requiring further research that considers the specific timing of physical activity in and around public transport trips throughout the day to disentangle this finding. This study strengthens the case for the promotion of public transport and the need for inter-sectoral action as important preventive strategies for improving public health.

### Electronic supplementary material

Below is the link to the electronic supplementary material.

Supplementary Material 1

## Data Availability

The datasets used and/or analysed during the current study are available from the corresponding author on reasonable request.

## Abbreviations

CI: Confidence interval
IQR: Inter quartile range
MVPA: Moderate-to-vigorous physical activity
SD: Standard deviation
STROBE: Strengthening the reporting of observational studies in epidemiology
