# Supplementary material for: Is public transport a promising strategy for increasing physical activity? Evidence from a study of objectively measured public transport use and physical activity
Source: Int J Behav Nutr Phys Act. 2024 Aug 19;21:91. doi: 10.1186/s12966-024-01633-3 (PMC11331653; doi:10.1186/s12966-024-01633-3)
Supplement: Supplementary file 1 — Supplementary Material 1 [file 12966_2024_1633_MOESM1_ESM.docx]

**SUPPLEMENTARY MATERIAL – STROBE Statement**

**Is public transport a promising strategy for increase physical activity? Evidence from a study of objectively measured public transport use and physical activity.**

Jack. T. Evans ^1^, Oliver Stanesby ^1, 2^, Leigh Blizzard ^1^, Stephen Greaves ^3^, Anna Timperio ^4^,

Kim Jose ^1^, Melanie J. Sharman ^1^, Andrew J. Palmer ^1^, Verity J. Cleland ^1, 4^

^1.^ Menzies Institute for Medical Research, University of Tasmania, Hobart, Australia

^2.^ Baker Heart and Diabetes Institute, Melbourne, Australia

^3.^ Institute of Transport and Logistics Studies, University of Sydney, Sydney, Australia

^4.^ School of Exercise and Nutrition Sciences, Deakin University, Geelong, Australia

| **Table S1.** STROBE Statement—Checklist of items that should be included in reports of *cohort studies* | | | |
| --- | --- | --- | --- |
|  | Item No | Recommendation | Page |
| **Title and abstract** | 1 | (*a*) Indicate the study’s design with a commonly used term in the title or the abstract | 1 |
|  |  | (*b*) Provide in the abstract an informative and balanced summary of what was done and what was found | 2 |
| Introduction | | |  |
| Background/rationale | 2 | Explain the scientific background and rationale for the investigation being reported | 4-5 |
| Objectives | 3 | State specific objectives, including any prespecified hypotheses | 5 |
| Methods | | |  |
| Study design | 4 | Present key elements of study design early in the paper | 5-6 |
| Setting | 5 | Describe the setting, locations, and relevant dates, including periods of recruitment, exposure, follow-up, and data collection | 5-6 |
| Participants | 6 | (*a*) Give the eligibility criteria, and the sources and methods of selection of participants. Describe methods of follow-up | 5-6 |
|  |  | (*b*) For matched studies, give matching criteria and number of exposed and unexposed | n/a |
| Variables | 7 | Clearly define all outcomes, exposures, predictors, potential confounders, and effect modifiers. Give diagnostic criteria, if applicable | 7-8 |
| Data sources/ measurement | 8* | For each variable of interest, give sources of data and details of methods of assessment (measurement). Describe comparability of assessment methods if there is more than one group | 7-8 |
| Bias | 9 | Describe any efforts to address potential sources of bias | 6-8 |
| Study size | 10 | Explain how the study size was arrived at | 6-8 |
| Quantitative variables | 11 | Explain how quantitative variables were handled in the analyses. If applicable, describe which groupings were chosen and why | 6-8 |

| **Table S1.** *continued*. STROBE Statement—Checklist of items that should be included in reports of *cohort studies* | | | | |
| --- | --- | --- | --- | --- |
| Statistical methods | | 12 | (*a*) Describe all statistical methods, including those used to control for confounding | 8-9 |
|  |  |  | (*b*) Describe any methods used to examine subgroups and interactions | 8-9 |
|  |  |  | (*c*) Explain how missing data were addressed | n/a |
|  |  |  | (*d*) If applicable, explain how loss to follow-up was addressed | n/a |
|  |  |  | (*e*) Describe any sensitivity analyses | 8 |
| Results | | | |  |
| Participants | 13* | (a) Report numbers of individuals at each stage of study—e.g., numbers potentially eligible, examined for eligibility, confirmed eligible, included in the study, completing follow-up, and analysed | | 6, 9  Fig.1 |
|  |  | (b) Give reasons for non-participation at each stage | | Fig.1 |
|  |  | (c) Consider use of a flow diagram | | Fig.1, |
| Descriptive data | 14* | (a) Give characteristics of study participants (e.g., demographic, clinical, social) and information on exposures and potential confounders | | 9, Tab.1 |
|  |  | (b) Indicate number of participants with missing data for each variable of interest | |  |
|  |  | (c) Summarise follow-up time (e.g., average and total amount) | | 6 |
| Outcome data | 15* | Report numbers of outcome events or summary measures over time | | 9-10, Tab.1&2  Fig 2 |
| Main results | 16 | (*a*) Give unadjusted estimates and, if applicable, confounder-adjusted estimates and their precision (eg, 95% confidence interval). Make clear which confounders were adjusted for and why they were included | | Tab.2  9-11 |
|  |  | (*b*) Report category boundaries when continuous variables were categorized | | n/a |
|  |  | (*c*) If relevant, consider translating estimates of relative risk into absolute risk for a meaningful time period | | n/a |
| Other analyses | 17 | Report other analyses done—e.g., analyses of subgroups and interactions, and sensitivity analyses | | 11 |

| **Table S2.** Distribution of daily trips by person and observation days at each timepoint | | | |
| --- | --- | --- | --- |
|  | **Trips/day (n)** | **Participants**, n (%) | **Observations**, days (%) |
| **Timepoint 1** |  | N=70 | N=489 |
|  | 0 | 70 (100.0) | 361 (73.8) |
|  | 1 | 35 (50.0) | 56 (11.4) |
|  | 2 | 42 (60.0) | 61 (12.5) |
|  | 3 | 6 (8.6) | 8 (1.6) |
|  | >3 | 3 (4.3) | 3 (0.7) |
| **Timepoint 2** |  | N=45 | N=308 |
|  | 0 | 45 (100.0) | 251 (81.5) |
|  | 1 | 13 (28.9) | 20 (6.5) |
|  | 2 | 16 (35.6) | 26 (8.5) |
|  | 3 | 3 (6.7) | 5 (1.6) |
|  | >3 | 3 (6.7) | 6 (1.9) |
| **Timepoint 3** |  | N=49 | N=339 |
|  | 0 | 48 (98.0) | 281 (82.9) |
|  | 1 | 13 (26.5) | 23 (6.8) |
|  | 2 | 18 (36.7) | 29 (8.5) |
|  | 3 | 2 (8.2) | 3 (0.9) |
|  | >3 | 2 (4.1) | 3 (0.9) |
| **Timepoint 4** |  | N=47 | N=333 |
|  | 0 | 46 (97.9) | 312 (93.7) |
|  | 1 | 8 (17.0) | 13 (3.9) |
|  | 2 | 6 (12.8) | 8 (2.4) |
|  | 3 | 0 (0.0) | 0 (0.0) |
|  | >3 | 0 (0.0) | 0 (0.0) |
| **Total** |  | N=73 | N=1483 |
|  | 0 | 73 (100.0) | 1219 (82.2) |
|  | 1 | 46 (63.0) | 112 (7.5) |
|  | 2 | 56 (76.7) | 124 (8.4) |
|  | 3 | 10 (13.7) | 16 (1.1) |
|  | >3 | 8 (11.0) | 12 (0.8) |
|  | | | |

| **Table S3.** Distribution of daily trips by person and observation days at each timepoint, stratified by treatment group. | | | | | |
| --- | --- | --- | --- | --- | --- |
|  |  | **Control Group** | | **Intervention Group** | |
|  | **Trips** (n) | **Participants**,  n (%) | **Observations**,  Days (%) | **Participants**,  n (%) | **Observations**,  days (%) |
| **Timepoint 1** |  | N=32 | N=220 | N=38 | N=269 |
|  | 0 | 32 (100.0) | 165 (75.0) | 38 (100.0) | 196 (72.9) |
|  | 1 | 13 (40.6) | 23 (10.4) | 22 (57.9) | 33 (12.3) |
|  | 2 | 19 (59.4) | 29 (13.2) | 23 (60.5) | 32 (11.9) |
|  | ≥3 | 3 (9.4) | 3 (1.4) | 5 (13.2) | 8 (3.0) |
| **Timepoint 2** |  | N=21 | N=138 | N=24 | N=170 |
|  | 0 | 21 (100.0) | 113 (81.9) | 24 (100.0) | 138 (81.2) |
|  | 1 | 5 (23.8) | 10 (7.3) | 8 (33.3) | 10 (5.9) |
|  | 2 | 7 (33.3) | 14 (10.1) | 9 (37.5) | 12 (7.0) |
|  | ≥3 | 1 (4.8) | 1 (0.7) | 5 (20.8) | 10 (5.9) |
| **Timepoint 3** |  | N=25 | N=167 | N=24 | N=172 |
|  | 0 | 24 (96.0) | 140 (83.8) | 24 (100.0) | 141 (82.0) |
|  | 1 | 6 (24.0) | 9 (5.4) | 7 (29.2) | 14 (8.2) |
|  | 2 | 10 (40.0) | 18 (10.8) | 8 (33.3) | 11 (6.4) |
|  | ≥3 | 0 (0.0) | 0 (0.0) | 4 (8.3) | 6 (3.5) |
| **Timepoint 4** |  | N=23 | N=163 | N=24 | N=170 |
|  | 0 | 23 (100.0) | 151 (92.6) | 23 (95.8) | 161 (94.7) |
|  | 1 | 5 (21.7) | 8 (4.9) | 3 (12.5) | 5 (2.9) |
|  | 2 | 4 (17.4) | 4 (2.5) | 2 (8.3) | 4 (2.4) |
|  | ≥3 | 0 (0.0) | 0 (0.0) | 0 (0.0) | 0 (0.0) |
| **Total** |  | N=33 | N=695 | N=40 | N=788 |
|  | 0 | 33 (100.0) | 576 (82.9) | 40 (100.0) | 643 (81.6) |
|  | 1 | 21 (63.6) | 50 (7.2) | 25 (62.5) | 62 (7.9) |
|  | 2 | 26 (78.8) | 65 (9.3) | 30 (75.0) | 59 (7.5) |
|  | ≥3 | 4 (12.1) | 4 (0.6) | 12 (30.0) | 24 (3.0) |
|  | | | | | |
